# Gastric Trichobezoars in paediatric population– A series of six cases and literature review

**DOI:** 10.1016/j.amsu.2022.104906

**Published:** 2022-11-13

**Authors:** Murad Habib, Muhammad Bin Amjad, Muhammad Abbas, Muhammad Amjad Chaudhary

**Affiliations:** a Department of Neonatal Paediatric Surgery, The Children's Hospital, Pakistan Institute of Medical Sciences, Islamabad, 44000, Pakistan; b Islamabad Medical and Dental College, Islamabad, 44000, Pakistan; c Riphah Institute of Pharmaceutical Sciences, Riphah International University Islamabad, 4400, Pakistan

**Keywords:** Gastric, Trichobezoars, Paediatric, Surgical exploration

## Abstract

**Background:**

Trichobezoar is a rare gastrointestinal pathology in paediatric population. Patients present with a range of symptoms from being asymptomatic to abdominal pain with a palpable abdominal mass. Once diagnosed it warrants urgent retrieval as delayed diagnosis may lead to serious complications.

**Methods:**

We present a series of six cases between March 2021 and March 2022 who presented to Children's Hospital and were diagnosed as a case of Gastric Trichobezoars. Patients were optimized and prepared for surgery. All patients underwent Surgical exploration and a tuft of hair were removed. They were followed up throughout the course of treatment and three of the patients underwent psychiatric evaluation.

**Discussion and conclusion:**

Trichobezoar is a rare but important surgical case that is a manifestation of underlying psychiatric ailment. Presentation varies from asymptomatic masses to life threatening complications with delayed presentations. A multi-disciplinary approach including Psychiatric, Paediatrician and Paediatric surgeon should be undertaken. Follow-up is the mainstay of treatment and recurrence may be seen due to non-compliance or inadequate management.

## Highlights

• Gastric trichobezoar is extremely rare in paediatric population.
• Patient presents with non-specific symptoms and can easily be missed.
• If a diagnosis of Gastric Trichobezoar is made, then it's a surgical emergency.
• If a Trichobezoar extends beyond pylorus into the small intestine it's called Rapunzel syndrome.
• Psychiatric evaluation should be done for underlying disorder (trichotillomania).

## 1 Introduction

Trichobezoar is a relatively rare (<1%) clinical entity in paediatric population. Its presentation varies from chronic abdominal pain, anorexia, weight loss, upper gastrointestinal symptoms to acute gastric outlet or intestinal obstruction in severe cases. Delayed diagnosis results in various complications [1].

Bezoars are the foreign bodies of Gastrointestinal tract that originate primarily in the stomach, consisting of non-digestive food particles which accumulate over time in the stomach [2]. These may consist of different compounds including vegetable fibers (phytobezoar), congealed milk or formulas (lactobezoar) and hair (trichobezoar).

Stomach has smooth surface hence it resists digestion as well as peristalsis of human hair. But if the person continues to ingest it becomes accumulated between the mucosal folds of the stomach. And combined with mucous secretion from stomach and food particles, it gives a mass like effect forming a gastric trichobezoar [3]. In most of the cases trichobezoar is confined within the stomach. In some cases, however, the trichobezoar extends through the pylorus into jejunum, ileum or even colon. This condition called Rapunzel syndrome first described by Vaughan et al., in 1968. Incidentally, parts of the tail can break off and migrate to the small intestine, causing intestinal obstruction [4].

Early presentation is rarely seen as the patient is completely asymptomatic to mild abdominal pain. So they continue on ingesting the hair until they developed severe abdominal pain along with obstructive symptoms. It is the time when that tuft of hair along with stomach contents has evolved into a large trichobezoar. In young females it is associated with psychiatric illness, as it is usually the result of the urge to pull out one's own hair (trichotillomania) and swallow it (trichophagia) [[5], [6]].

The aim of presenting our case series is to share our experience in the management of trichobezoars in paediatric population. Records of six patients who visited our outpatient and emergency department from March 2021 to March 2022 was gathered. It is interesting to see variety of symptoms they present with, some completely unaware of the condition, others with abdominal pain and mass while completely different picture in the emergency department where they present with acute abdomen. All the patients were treated surgically and a trichobezoar was removed, psychiatric evaluation was taken for the patients where necessary.

## 2 Materials and methods

We present a series of 6 cases between March 2021 and March 2022 who presented to Children's Hospital under the Ethical approval #F.1-1/2015/ERB/SZBMU/642, according to the PROCESS Guidelines [7]. They were diagnosed as a case of Gastric Trichobezoars.

All patients were optimized prior to surgery underwent Surgical exploration and a tuft of hair were removed.

Inclusion criteria: All patients with an established diagnosis of gastric Trichobezoar were included in the study younger than 13 years of age.

Exclusion criteria: Patients older than 13 years with a diagnosis of gastric Trichobezoar were excluded from the study.

## 3 Case series

### 3.1 Case 01

**Fig. 1:**
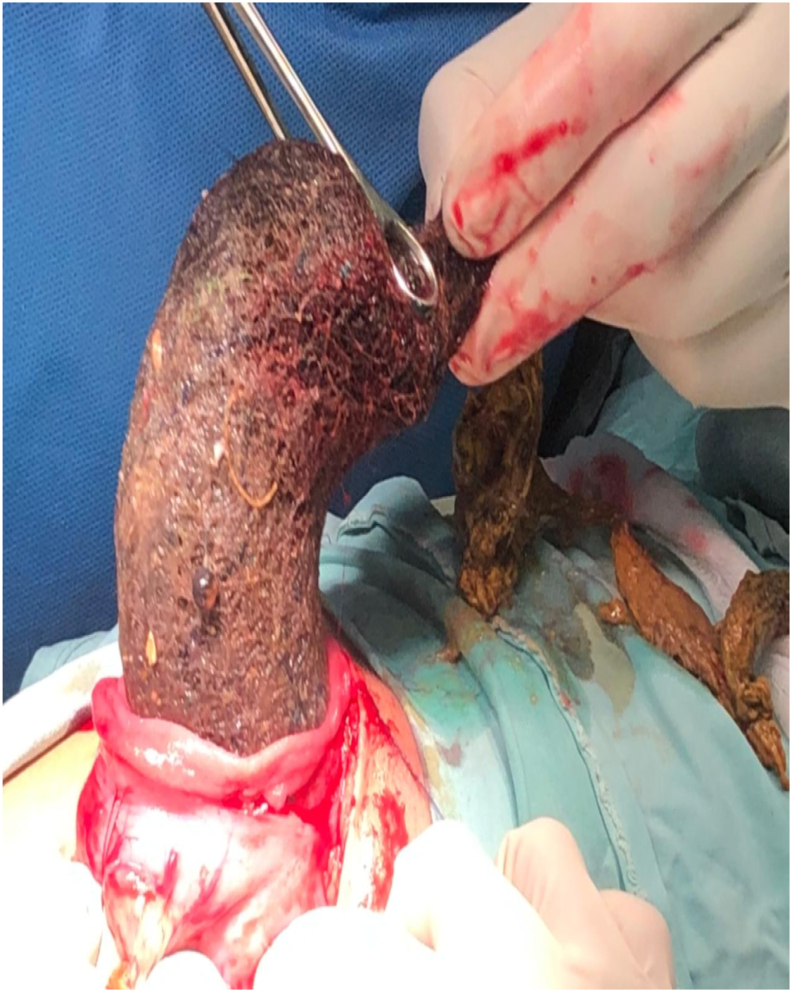
Intragastric Trichobezoar.

A 9-year-old female child presented via outdoor patient with a history of abdominal pain and weight loss since 2 months and an upper abdominal mass noted by the mother. On examination, she had strikingly thin hair on the scalp and a palpable non tender, mobile mass in the epigastric region measuring 7 × 8 cm approximately. X-ray abdomen/pelvis was unremarkable. On Ultrasound abdomen no mass noted. Mild pelvic ascites. CT Scan Abdomen and Pelvis with IV Contrast showed well defined, in-homogenous, intra-gastric mass measuring 4x6x11 cm with mottled gas pattern with body in gastric fundus and tail in pylorus with extension into duodenum. The patient was admitted to surgical ward and resuscitated. He was administered with intravenous fluids and broad spectrum antibiotics pre-operatively. He was shifted to operation theater and prepared for surgery. Exploratory Laparotomy was performed. Mass of hairs mixed with food particles was found in stomach. Gastrotomy and retrieval was done (Fig. 1). Patient followed swift post-operative recovery and was discharged on fifth postoperative day along with psychiatrist evaluation.

### 3.2 Case 02

A 12-year-old female child presented via OPD with history of anorexia, weight loss since 6 months and progressively worsening nausea and vomiting since 1 month. On examination, she had a palpable non tender, mobile mass in the epigastric region measuring 8 × 8cm approximately. X-ray abdomen/pelvis was unremarkable. CT Scan Abdomen and Pelvis with IV Contrast showed well defined, inhomogeneous, intra-gastric mass measuring 3.7 x 9.3 × 13.5 cm with mottled gas pattern with body in gastric fundus and tail in pylorus with extension into duodenum. The patient was admitted to surgical ward and resuscitated. He was administered with intravenous fluids and broad spectrum antibiotics pre-operatively. He was shifted to operation theater and prepared for surgery Exploratory Laparotomy was performed. Mass of hairs mixed with food particles was found in stomach, reaching up to the Jejunum. Gastrotomy and retrieval was done. The patient's post-operative recovery was unremarkable and psychiatrist evaluation was taken.

### 3.3 Case 03

A 6-year-old female child presented to emergency department with complaints of billious vomiting, abdominal distension, and absolute constipation for 3 days. On examination her abdomen was distended with mobile mass palpable in the right lower quadrant. Digital rectal examination showed scanty stool in rectum. Xray Abdomen/Pelvis showed dilated small bowel loops with scanty air in pelvis. The patient was admitted to surgical ward and resuscitated. He was administered with intravenous fluids and broad spectrum antibiotics pre-operatively. He was shifted to operation theater and prepared for surgery. Trichobezoar causing bolus obstruction at distal ileum was noted. Bezoar milked into colon, no enterotomy was performed. Patient followed a swift post-operative recovery.

### 3.4 Case 04

**Fig. 2:**
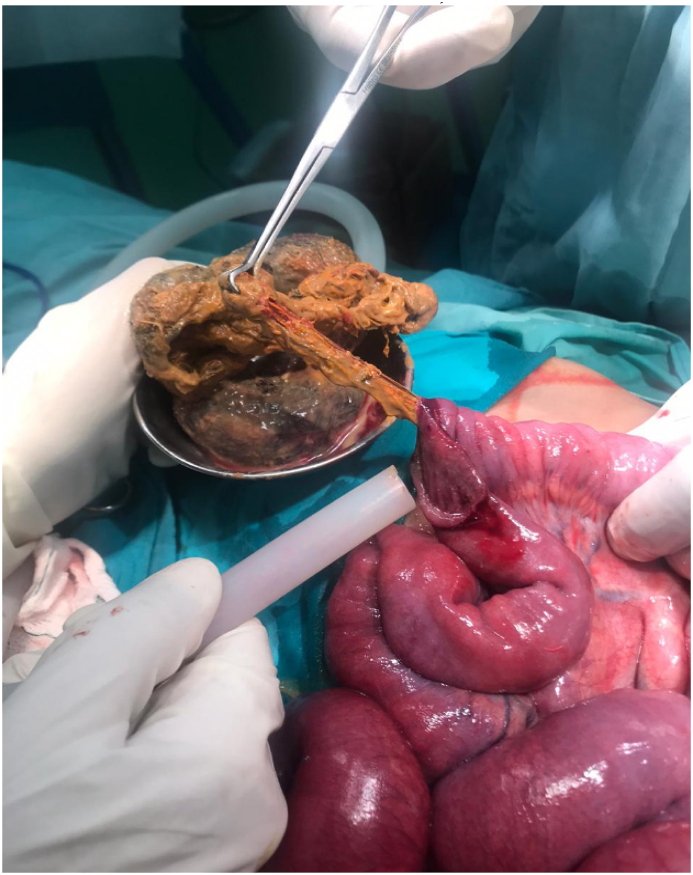
Trichobezoar being removed from Proximal Ileum.

A 6-year-old female child presented to emergency department with complaints of bilious vomiting, abdominal distension, and constipation. On examination her abdomen was tender, distended with mobile mass palpable in the right lower quadrant. DRE showed empty rectum. X-ray Abdomen/Pelvis showed dilated jejunal loops with scanty air in pelvis. The patient was admitted to surgical ward and resuscitated. He was administered with intravenous fluids and broad spectrum antibiotics pre-operatively. He was shifted to operation theater and prepared for surgery. Trichobezoar causing bolus obstruction at proximal ileum was noted. Enterotomy and retrieval was done (Fig. 2). Recovery was unremarkable.

### 3.5 Case 05

**Fig. 3:**
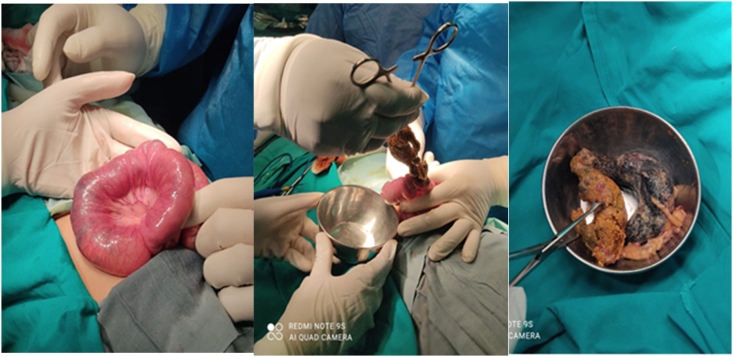
Trichobezoar being retrieved from mid-ileum.

A 10-year-old female child presented to emergency department with complaints of bilious vomiting, abdominal pain and distension, constipation. Her abdomen was distended with mobile mass right lower quadrant. Xray Abdomen/Pelvis showed dilated small bowel loops with scanty air in pelvis. Ultrasound Abdomen/Pelvis showed heterogeneous lesion in right hypochondrium measuring 28 × 33mm giving target sign, likely representing intussusception. The patient was admitted to surgical ward and resuscitated. He was administered with intravenous fluids and broad spectrum antibiotics pre-operatively. He was shifted to operation theater and prepared for surgery. Exploratory Laparotomy was performed and a Trichobezoar causing bolus obstruction at mid ileum was noted. Enterotomy and retrieval was done (Fig. 3).

## 3.6 Case 6

A 12-year-old Male child presented via emergency with history of severe abdominal pain, vomiting and abdominal distension. He was previously operated two years back for gastric trichobezoar, Elective laparotomy and gastrotomy for retrieval was performed. Child was started on behavior modification therapy and TCAs however there was noncompliance on the part of parents and psychiatrist follow up was sought. On examination, he was a sick, toxic looking child with distended abdomen and tenderness all over. X-ray abdomen/pelvis showed pneumo-peritoneum. The patient was admitted to surgical ward and resuscitated. He was administered with intravenous fluids and broad spectrum antibiotics pre-operatively. He was shifted to operation theater and prepared for surgery. Operative findings were gastric perforation noted at the anterior surface of gastric body. Trichobezoar occupying entirety of stomach and extending into proximal small bowel, this was evacuated. Margins of perforation refreshed and repaired. Patient's post-operative recovery was un-eventful. All the characteristics are shown in Table 1.

**Table 1:** Summary of case series of Gastric Trichobezoars in paediatric population.

Cases	Age	Gender	Symptoms	Duration	Mode of presentation	Diagnosis	ASA	Outcome	Psychiatric illness
1	9 years	Female	Abdominal pain, Loss of weight	2 months	Emergency	Gastric trichobezoar	ASA-2	Exploratory Laparotomy plus gastrotomy	Yes
2	12 years	Female	Anorexia, Weight loss	6 months	Emergency	Gastric trichobezoar	ASA-3	Exploratory Laparotomy plus gastrotomy	Yes
3	6 years	Female	Billious vomiting, abdominal distension, constipation	3 days	Emergency	Gastric trichobezoar	ASA-2	Exploratory Laparotomy plus enterotomy (jejunum)	No
4	6 years	Female	Billious vomiting, abdominal distension, constipation	7 days	Emergency	Gastric trichobezoar	ASA-2	Exploratory Laparotomy plus enterotomy (proximal ileum)	No
5	10 years	Female	Vomiting, abdominal pain and distension	15 days	Emergency	Gastric trichobezoar	ASA-2	Exploratory Laparotomy plus enterotomy (mid ileum)	No
6	12 years	Male	Abdominal pain, Vomiting, distension	1 year	Emergency	Gastric trichobezoar	ASA-3	Exploratory Laparotomy plus gastrotomy	Yes

## 4 Discussion

Trichobezoar is a rare obstructive condition with increased prevalence in young females between the ages of 10–19 years who display an increased predisposition, having a history of the psychiatric illness trichotillomania (patient removes their own hair) and trichophagia (ingests the hair) as the primary psychiatric disorder which may be associated with underlying disorders such depression, obsessive-compulsion disorder, pica and anorexia nervosa [7]. A widely accredited hypothesis on their formation proposes that the indigestible and slippery nature of the surface of hair fibers prevents propulsion by peristalsis resulting in their retention within the gastric mucosal folds [3]. As hair further accumulates it is entangled until it forms a ball of significant size which cannot exit the stomach resulting in gastric atony. These large masses of hair become further intertwined with one another to form a single mass that is molded into the shape of the stomach [3,8]. The mucus covering the mass gives it a glistening and smooth surface, with the decomposition and fermentation of fats giving it the characteristic rancid smell [9].

Initially asymptomatic during the earlier stages persistent collection of hair increases obstruction, resulting in a presentation of malaise, vomiting, generalized abdominal pain and anorexia over time resulting in loss of weight and cachexia [3]. Eventually over time, the trichobezoar will extend into the small intestine as a tail, a condition called Rapunzel syndrome, reported initially by Vaughan and colleagues in 1963 [10].

Rapunzel syndrome is a rare progression of trichobezoar as over time the accumulation of hair will cause the trichobezoar to grow beyond the stomach and into the small intestine, over the decades various criteria have been utilised in literature to describe the condition, Descriptions include a tail extending up to jejunum and further beyond [11,12].

As the patient presents with non-specific symptoms radiology plays an important in diagnosing the underlying pathology including abdominal radiograph which shows a distended stomach, Ultrasound sensitivity, specificity which may reveal highly echogenic areas and the mass casting a significantly dense sonic shadow, barium studies also reveal an inter gastric-mass with barium in the interstices of the mass [13,14]. Computer tomography has sensitivity of scan of abdomen is more definitive than ultrasound and reveals a well-defined intraluminal mass of consisting of concentric whorls with air enmeshed in the interstices and may reveal presence of dilated intestinal loops (rapunzel syndrome) [14]. However upper GI endoscopy is considered to be the best diagnostic tool [15].

Different treatment modalities currently exist for management of trichobezoars depending upon the availability and access to endoscopic facilities at the hospital.

Irrespective of facilities available a gastric trichobezoar warrants an urgent removal once diagnosed if no intervention is made the mass may erode into the gastric mucosa causing ulceration, hemorrhage and perforation [3].

Although Endoscopy has been used increasingly over the past decade but it is not a viable option for larger trichobezoars and repeatedly performing this procedure can lead to devastating complications like esophageal perforation [15,16].

Laparoscopy is more effective than endoscopy while having less complications compared to laparotomy. The bezoars is first fragmented into pieces by polypectomy snare and argon plasma coagulation and then removed piece by piece [17,18].

Exploration by Laparotomy is considered to be the most effective treatment for trichobezoars having the highest success rate, and the gold standard for large trichobezoars which may extend beyond the pylorus such in the cases of Rapunzel syndrome where a trichobezoar can extend to small intestine and even to ileo-cecal valve where an enterotomy and retrieval is done [11].

A retrospective study by Gorter et al. on 108 patients compare different treatment modalities, endoscopic treatment 5%, laparoscopic approach 75% and laparotomy a 100% success rate which is also preferred in complicated cases [19].

The surgical treatment of the trichobezoars is however not the only element, as the underlying psychiatric disorder is the causative factor in the development of trichobezoars and psychiatric care makes an important part of the management as one of our patient was on tricyclic anti-depressants. Thus a multi-disciplinary approach is the mainstay of treatment [20].

Prevention is better than cure and correction of the psychiatric illness will not suffice alone if environmental and social stressors that precipitated the trichophagia are not treated with a holistic approach which would utilize cognitive behavioral therapy among other forms of psychotherapy, antidepressant and antipsychotic medications combining all these treatment modalities to achieve successful recovery [21].

## 5 Conclusion

Trichobezoar is a rare but important surgical case that is a manifestation of underlying psychiatric ailment. Presentation varies from asymptomatic masses to life threatening complications with delayed presentations. A multi-disciplinary approach including Psychiatric, Paediatrician and Paediatric surgeon should be undertaken. Follow-up is the mainstay of treatment and recurrence may be seen due to non-compliance or inadequate management.

## Availability of data and materials

All data can be made available upon request to the corresponding authors.

## Ethical approval

This study was reviewed and approved by ethical review board committee of Pakistan Institute of Medical Sciences, Islamabad, Pakistan (F.1-1/2015/ERB/SZBMU/642) and all methods were carried out in accordance with relevant guidelines and regulations.

## Sources of funding

Not available.

## Authors contribution

Concept of study: Murad habib, Muhammad Abbas, Muhammad Amjad Chaudhary.

Acquisition of data: Murad Habib, Muhammad bin Amjad.

Interpretation of data: Murad Habib, Muhammad abbas.

Intellectual content: Murad Habib, Muhammad bin Amjad, Muhammad Abbas.

Supervision: Muhammad Amjad Chaudhary.

## Registration of research studies

1. Name of the registry: Research registry
2. Unique Identifying number or registration ID: researchregistry8371
3. Hyperlink to your specific registration (must be publicly accessible and will be checked): https://www.researchregistry.com/register-now#home/registrationdetails/6339458043cd220021d3139f/

## Guarantor

Muhammad Amjad Chaudhary.

## Consent

All participants provided informed consent prior to study participation. Informed consent was obtained from the corresponding parent (male or female), and they were free to decline/withdraw from the study at any point.

## Declaration of competing interest

None declared.

## References

[1] Gorter R.R., Kneepkens C.M., Mattens E.C., Aronson D.C., Heij H.A. (2010 May). Management of trichobezoar: case report and literature review. Pediatr. Surg. Int..

[2] Eng K., Kay M. (2012 Nov). Gastrointestinal bezoars: history and current treatment paradigms. Gastroenterol. Hepatol..

[3] Pace AM, Fearne C. Trichobezoar in a 13-Year-Old Male: a Case Report and Review of Literature.

[4] Gupta A., Gupta P.L. (2020 Apr 23). Gastric trichobezoar and Rapunzel syndrome: a case report. Int. J. Surg..

[5] Grant J.E., Chamberlain S.R. (2016 Sep 1). Trichotillomania. Am. J. Psychiatr..

[6] Gorter R.R., Kneepkens C.M., Mattens E.C., Aronson D.C., Heij H.A. (2010 May). Management of trichobezoar: case report and literature review. Pediatr. Surg. Int..

[7] Agha R.A., Sohrabi C., Mathew G., Franchi T., Kerwan A., O'Neill N. (2020). The PROCESS 2020 guideline: updating consensus preferred reporting of CasE series in surgery (PROCESS) guidelines. Int. J. Surg..

[8] Sood A.K., Bahl L., Kaushal B.K. (2000). Childhood trichobezoar. Indian J. Pediatr..

[9] Sidhu B.S., Singh G., Khanna S. (1993 Apr 1). Trichobezoar. J. Indian Med. Assoc..

[10] Vaughan E.D., Sawyers J.L., Scott H.W. (1968 Feb). The Rapunzel syndrome. An unusual complication of intestinal bezoar. Surg..

[11] Naik S., Gupta V., Naik S., Rangole A., Chaudhary A.K., Jain P., Sharma A.K. (2007). Rapunzel syndrome reviewed and redefined. Dig. Surg..

[12] Gupta A., Gupta P.L. (2020 Apr 23). Gastric trichobezoar and Rapunzel syndrome: a case report. Int. J. Surg..

[13] Ratcliffe J.F. (1982 Feb). The ultrasonographic appearance of a trichobezoar. Br. J. Radiol..

[14] Dogra S., Kulkarni A.K., Rao P.P. (2012 Jul). Rapunzel syndrome—a case report. Med. J. Armed Forces India.

[15] Al-Osail E.M., Zakary N.Y., Abdelhadi Y. (2018 Jan 1). Best management modality of trichobezoar: a case report. Int. J. Surg. Case Rep..

[16] Kanetaka K., Azuma T., Ito S., Matsuo S., Yamaguchi S., Shirono K., Kanematsu T. (2003 Feb 1). Two-channel method for retrieval of gastric trichobezoar: report of a case. J. Pediatr. Surg..

[17] Khirallah M.G., El-Dessouki N.I., Kurdi M. (2022 Mar 22). Laparoscopic management of gastric trichobezoar in children: a case series study. J. Pediatr. Endosc. Surg..

[18] Kim S.C., Kim S.H., Kim S.J. (2016 May). A case report: large trichobezoar causing rapunzel syndrome. Med..

[19] Gorter R.R., Kneepkens C.M., Mattens E.C., Aronson D.C., Heij H.A. (2010 May). Management of trichobezoar: case report and literature review. Pediatr. Surg. Int..

[20] Ali A.A., Gurung R., Fuad Z.M., Moosa M., Ali I., Abdulla A., Muhamad A., Hayati F., Pang N.T. (2020 Oct 1). Gastric trichobezoar in an end-stage renal failure and mental health disorder presented with chronic epigastric pain: a case report. Ann. Med. Surg..

[21] Pinto A.C., Andrade T.C., Brito F.F., Silva G.V., Cavalcante M.L., Martelli A.C. (2017 Jan). Trichotillomania: a case report with clinical and dermatoscopic differential diagnosis with alopecia areata. An. Bras. Dermatol..

